# Explicitly encoding the cyclic nature of breathing signal allows for accurate breathing motion prediction in radiotherapy with minimal training data

**DOI:** 10.1016/j.phro.2024.100594

**Published:** 2024-05-27

**Authors:** Andreas Renner, Ingo Gulyas, Martin Buschmann, Gerd Heilemann, Barbara Knäusl, Martin Heilmann, Joachim Widder, Dietmar Georg, Petra Trnková

**Affiliations:** a Department of Radiation Oncology, Medical University of Vienna, Vienna, Austria; b Christian Doppler Laboratory for Image and Knowledge Driven Precision Radiation Oncology, Department of Radiation Oncology, Medical University of Vienna, Austria; c MedAustron Ion Therapy Center, Wiener Neustadt, Austria

**Keywords:** Real-time tumour motion monitoring, Motion prediction, Intrafractional motion, 4D image guidance, Long short-term memory network

## Abstract

**Background and purpose:**

Active breathing motion management in radiotherapy consists of motion monitoring, quantification and mitigation. It is impacted by associated latencies of a few 100 ms. Artificial neural networks can successfully predict breathing motion and eliminate latencies. However, they require usually a large dataset for training. The objective of this work was to demonstrate that explicitly encoding the cyclic nature of the breathing signal into the training data enables significant reduction of training datasets which can be obtained from healthy volunteers.

**Material and methods:**

Seventy surface scanner breathing signals from 25 healthy volunteers in anterior-posterior direction were used for training and validation (ratio 4:1) of long short-term memory models. The model performance was compared to a model using decomposition into phase, amplitude and a time-dependent baseline. Testing of the models was performed on 55 independent breathing signals in anterior-posterior direction from surface scanner (35 lung, 20 liver) of 30 patients with a mean breathing amplitude of (5.9 ± 6.7) mm.

**Results:**

Using the decomposed breathing signal allowed for a reduction of the absolute root-mean square error (RMSE) from 0.34 mm to 0.12 mm during validation. Testing using patient data yielded an average absolute RMSE of the breathing signal of (0.16 ± 0.11) mm with a prediction horizon of 500 ms.

**Conclusion:**

It was demonstrated that a motion prediction model can be trained with less than 100 datasets of healthy volunteers if breathing cycle parameters are considered. Applied to 55 patients, the model predicted breathing motion with a high accuracy.

## Highlights

• Explicit encoding of breathing periodicity allows for training data size reduction.
• Volunteer-based training breathing signals used for motion prediction in patients.
• Training dataset of seventy volunteers was sufficient.
• A root-mean square error of 0.16 mm within 500 ms prediction horizon achieved in patients.
• Feasible approach towards an institution-specific prediction model.

## 1 Introduction

The treatment of tumours located in the thorax and abdomen are impacted by breathing motion [1], [2], [3]. To mitigate the impact of breathing motion, tumour motion management (TMM) is required during radiotherapy [4], [5]. In passive TMM, the planning target volume (PTV) encompassing all possible tumour positions visible on 4D computer tomography (CT) is defined, using either an internal target volume or statistical margin recipes [6]. An alternative is 4D robust optimization [7]. Passive TMM leads to irradiation of larger healthy tissue volumes and therefore higher probability of radiation-induced side effects. Moreover, these techniques fail if the motion amplitude is larger than the margin. Active TMM methods aim to anticipate breathing-induced tumour motion, enabling reduction of treatment margins and more conformal dose delivery with minimised dose to healthy tissue [8].

Active TMM consists of (1) monitoring the tumour motion with online in-room imaging [9]; (2) quantifying the motion with dedicated software [10] and (3) mitigating the motion using either tracking [11], [12], [13], gating [14], [15], [16] or breath-hold techniques [17], [18]. Each of these steps is afflicted with system latencies of up to 500 ms related to image acquisition, data transfer, computation time and mechanical multi-leaf collimator (MLC) movement [19], [20], [21], [22], [23], [24]. The reduction of the system latencies is important especially for tumour tracking, where the MLC follows the tumour position in real-time and gating where the beam is turned on and off.

To visualize the internal tumour position during the treatment, online imaging with X-ray fluoroscopy or cine MR has been explored [9], [25], [26], both performed either continuously or at fixed time intervals. An alternative could be an approach that we refer to as *smart* imaging, where instead of imaging at fixed time intervals, a position verification image at the predicted position of the breathing phase is acquired. This will not only contribute to overcome latency effects and therefore enable real-time tumour tracking / gating, but also reduce imaging dose in case of online monitoring with X-ray. Reliable motion prediction is usually based on a continuous breathing motion surrogate signal [27]. Such a surrogate signal can be obtained from a surface scanner offering markerless real-time TMM with high frequency using non-ionising optical imaging [28], [29], [30]. As surface imaging performs comparably well to traditional X-ray based positioning [31], it was quickly adopted for image-guided radiotherapy and is widely available [32], [33].

Motion prediction can be performed using classic models like linear regression [34], [35], [36]. Nevertheless, artificial neural networks have been proven to outperform classic approaches [37]. Especially long short-term memory (LSTM) models seem to be well-suited for breathing motion prediction [38]. Lin *et al* [39] demonstrated on 1703 respiratory datasets from 985 patients across three institutions, acquired with an real-time position system, that tuning the hyper-parameters for training the model yielded a root mean squared error (RMSE) of 0.14 ± 0.09. The RMSE was reported relative to a normalised breathing amplitude ranging from [-1,1] for a prediction horizon of 500 ms representing the potential length of latencies.

Collecting such large datasets makes the development of an institution-specific model challenging, requiring several years for an average hospital to collect such an amount of high-quality data [40]. Alternatively, a large database collecting data from several institutes could speed up the process and provide more data variety, however data sharing is challenging due to patient privacy issues [41].

The objective of this work was to prove that an LSTM model can provide an accurate breathing motion prediction with a small training dataset obtained from healthy volunteers, by explicitly encoding the cyclic nature of the breathing signal into the training data. Testing with lung and liver patients using a prediction horizon of 500 ms was performed to demonstrate the clinical applicability.

## 2 Material and methods

### 2.1 Breathing motion data

The study was approved by the Institutional Review Board of Medical University of Vienna (Ethics Committee number EK-Nr. 1239/2022). Volunteers provided an informed consent. The patient data was collected retrospectively.

For training and validation of an LSTM model, 70 breathing motion datasets from 25 healthy volunteers (15 male, 10 female, 2–8 datasets/volunteer) were acquired with a Catalyst HD surface scanner (C-Rad, Sweden). The volunteers were positioned on the table of an Elekta Versa HD linear accelerator. No markers were used. Two surrogate surface locations (15 mm radius) at the sternum’s lower end (signal 1) and at the upper abdomen 2 cm above the navel (signal 2) were continuously scanned in anterior-posterior direction using free-breathing mode. Thus, two breathing signals with different characteristics were obtained within one acquisition. Prior to each acquisition, the volunteers were instructed to perform 5 min of regular breathing followed by 1 min of pure chest and 1 min of pure abdominal breathing. No dedicated breathing training was followed.

Testing of the best model was performed with 55 independent breathing motion datasets (35 lung, 20 liver) from 30 patients (15 lung, 15 liver, 1–6 datasets / patient). The eligibility criteria radiotherapy with image-guidance using optical surface scanning. Patients with less than 2 min of continuous undisturbed breathing signal were excluded. As breathing signal disturbance, table movement or talking were considered, but not coughing, irregular breathing, or sudden deep inspirations or expirations.

The average time per dataset was 4 min (range 2–10 min) yielding a total of 240 min of breathing data after pre-processing. The patient breathing signal was acquired with only one breathing surrogate point usually located right above the tumour centre. Sentinel surface scanner (C-rad, Sweden) at 4D-CT or the Catalyst surface scanner (C-Rad, Sweden) in the treatment room were used for signal acquisition.

### 2.2 Data pre-processing

The mean surface data acquisition frequency of the Catalyst was (13.3 ± 0.9) Hz for and (23.9 ± 1.2) Hz for the Sentinel. Due to these fluctuations, all datasets were interpolated to a constant acquisition frequency of 20 Hz. Additionally, all datasets were cropped to remove potential setup artefacts at the start and at the end of the acquisition process. To reduce noise, signal filtering through a lowpass filter using FilterDesigner in Matlab (R2019.b) was applied. For a maximally flat design, numerator and denominator order of 8 and a cut-off frequency of 1 Hz were selected, ensuring the effective attenuation of high-frequency noise while preserving the integrity of the breathing signal.

**Fig. 1:**
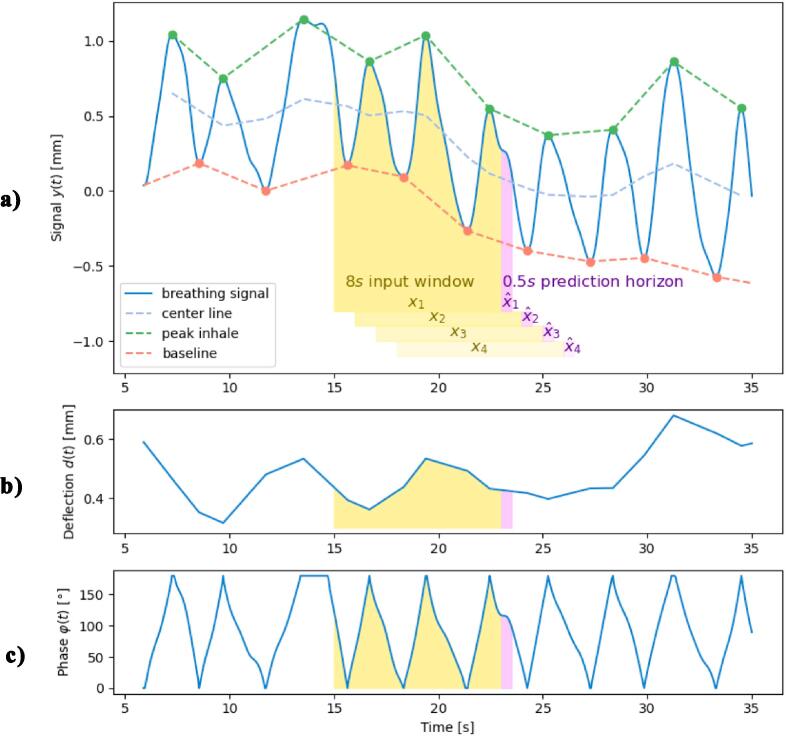
Demonstration of data pre-processing for Model 2. a) the interpolated and filtered dataset after peak-detection including the dynamically scaled baseline (dashed red line) and the centre line at 50 % inhale (dashed blue line) representing y_50%_ for dynamic scaling. The peaks were the basis for calculation of the deflection d shown in b) representing one-half of a breathing amplitude and the breathing phase φ(t) shown in c). Each prediction was based on the breathing signal of an input window x. The window length was a parameter of the LSTM model (illustrated with 8 s corresponding to the best results, see Section 3.2). The prediction horizon $\hat{x}$ was set at 500 ms.

All data gathered from healthy volunteers were used for development of two different LSTM models: a conventional model (Model 1) based on the breathing signal itself and a novel model where the cyclic nature of the breathing signal was explicitly encoded (Model 2). This was achieved by decomposing the breathing signal $y\left(t\right)$ into breathing phase $\varphi \left(t\right)$ and deflection $d\left(t\right)$ plus a time-dependent baseline $BL\left(t\right)$ (Fig. 1) using a modulated cosine function. For compact notation of $y\left(t\right)$ given in Equation (1), the deflection $d\left(t\right)$ was defined as one-half of the breathing amplitude (from max inspiration to max expiration):

(1) $$y\left(t\right)=d\left(t\right)+d\left(t\right)\cos\varphi \left(t\right)+BL\left(t\right)$$

The signal peaks of exhalation and inhalation were detected with the peakfinder function in Matlab 2019b using a threshold of 55 % of the mean breathing amplitude as this value gave the best results of peak detection (Fig. 1a). An exhalation peak was defined as 0° and a subsequent inhalation peak as 180°. Linear interpolation between peaks was performed to acquire the baseline and peak inhalation curves. The central value ($y_{50\%}$ at 50 % inhalation) was used to assess the deflection $d\left(t\right)$ of the model function and to measure the phase angle $\varphi \left(t\right)$ by transforming Equation (1).

Dynamic scaling was used for pre-processing of training data to enhance the influence of each breathing pattern segment, which was transformed by the scaling step into the range [−1,1]. The length of the segment was set with the input window parameter of the LSTM model (Fig. 1). The maximum $y_{\max}$ and minimum $y_{\min}$ of the input window were used to calculate the central value $y_{50\%}$ (Fig. 1a). The transformation of the unscaled breathing signal $y\left(t\right)$ into the scaled representation for LSTM model training followed Equation (2) for Model 1 and Equation (3) for Model 2.

(2) $$y_{\mathrm{scaled}}\left(t\right)=\frac{y\left(t\right)-y_{\min}-y_{50\%}}{y_{50\%}}$$

(3) $$y_{\mathrm{scaled}}\left(t\right)=\frac{d\left(t\right)}{y_{50\%}}-\frac{d\left(t\right)*\cos\varphi \left(t\right)}{y_{50\%}}+\frac{BL\left(t\right)-y_{\min}-y_{50\%}}{y_{50\%}}$$

### 2.3 Neural network architecture and model development

Model 1 was trained to predict the breathing signal $\hat{y}\left(t\right)$ (one output channel) from the breathing signal $y\left(t\right)$ of an input window x for a prediction horizon of 500 ms. Model 2 was trained to predict the breathing phase $\hat{\varphi }\left(t\right)$, deflection $\hat{d}\left(t\right)$, and baseline $\hat{\mathrm{BL}}\left(t\right)$ (three output channels) and the predicted breathing signal $\hat{y}\left(t\right)$ was reassembled from the three output channels using Equation (1).

The data of the healthy volunteers were divided in a ratio of 4:1 between training and validation. The architecture of both LSTM models consisted of three LSTM layers, with [40], [30], [20] units, followed by a dense layer for prediction output. The dataset was shuffled at the beginning of each training epoch, with 300 epochs in total. The model's hyper-parameters were chosen based on a range defined in Lin *et al* [39]. The input windows (in literature sometimes referred to as ‘time lag’) were set at 20, 40, 80, 160, 240 and 320 data points corresponding to 1 s, 2 s, 4 s, 8 s, 16 s and 32 s, respectively, at a breathing signal acquisition frequency of 20 Hz. The number of units in each LSTM layer was tested at 40, 30, and 20. The learning rate varied between 0.05, 0.01, 0.005, and 0.001. A fixed batch size of 512 was chosen to ensure a low noise level in the loss function. The code is published on GitHub (https://github.com/AndreasRenner/BreathingMotionPredictionLSTM).

For validation, the accuracy of the predicted breathing signal $\hat{y}\left(t\right)$ was evaluated using RMSE compared to the breathing signal $y\left(t\right)$ of the separate set of healthy volunteer breathing data not included in the training set.

The training was performed on a GPU Server having an Nvidia GeForce RTX 3080 with 10 GB of GPU memory, an Intel Core i9 CPU-10900 K CPU @ 3.70 GHz and 32 GB of RAM. As software framework Python 3.8, Tensorflow 2.6.2 and Keras 2.6.0 were used.

For runtime measurements, 100 consecutive predictions were performed on both, GPU and CPU, and the mean prediction time was measured.

### 2.4 Model testing

The model with the lowest absolute RMSE was validated with the patient datasets. The predicted values of $\hat{\varphi }\left(t\right)$, $\hat{d}\left(t\right)$, and $\hat{\mathrm{BL}}\left(t\right)$ as well as the reassembled breathing signal $\hat{y}\left(t\right)$ were compared against the true values from the patient dataset. The prediction accuracy was quantified in terms of absolute RMSE of $\varphi \left(t\right)$, $d\left(t\right)$, and $BL\left(t\right)$ and signal $y\left(t\right)$. To highlight the applicability of the model to patients with large breathing motions, the datasets were divided in two groups with a ‘small’ (presence of an amplitude ≤ 12 mm) and a ‘large’ (presence of an amplitude > 12 mm) breathing amplitude. For each group, the absolute RMSE was evaluated separately.

## 3 Results

### 3.1 Breathing motion data

**Fig. 2:**
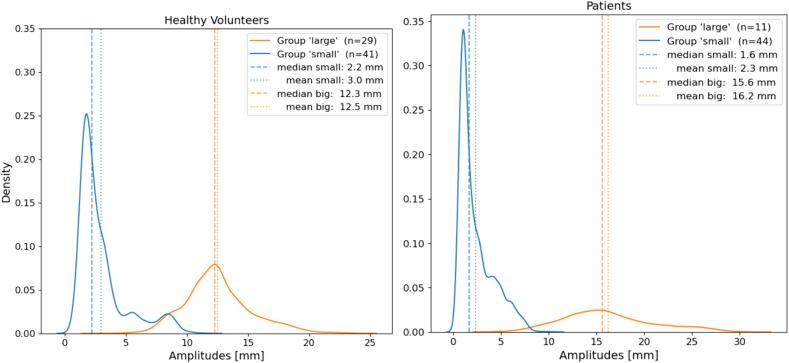
Distribution of all breathing amplitudes presented in the datasets. For both datasets (healthy volunteers on the left side and patients on the right side) the distribution of breathing amplitudes was separated into two groups: a group with a large breathing amplitude and a group with a small breathing amplitude where a presence of an amplitude > 12 mm was used for separation. The dashed and dotted vertical lines show the median and the mean of each group for each dataset, respectively.

Table 1 presents an overview of healthy volunteers and patients. In healthy volunteers, the mean breathing amplitude acquired from on the upper abdomen was more than four times bigger than from the sternum. The breathing amplitudes’ distribution are shown in Fig. 2. Twenty-nine (41 %) healthy volunteers and eleven (20 %) patients were assigned to ‘large’ group. All breathing signal acquisitions in the ‘large’ group of healthy volunteers were obtained from the abdominal signal.

**Table 1:** Breathing data statistics of volunteers and patients. For the healthy volunteers two signal positions were recorded: signal 1 on the lower part of the sternum and signal 2 on the upper abdomen; for the patients only one breathing signal was recorded usually located right above the tumour centre.

	datasets	duration [min]	min/max signal [mm]	mean amplitude [mm]
Healthy volunteers	All (n = 70)	266	−10.0/18.2	6.9 ± 5.2
	Signal 1 (sternum) (n = 35)	133	−7.2/6.5	2.4 ± 1.9
	Signal 2 (abdomen) (n = 35)	133	−10.0/18.2	10.7 ± 2.7
Patients	All (n = 55)	240	−11.5/19.9	5.9 ± 6.7
	‘Small’ amplitude (n = 44)	179	−5.5/7.3	2.3 ± 1.8
	‘Large’ amplitude (n = 11)	61	−11.5/19.9	16.2 ± 4.7

### 3.2 Model development

For Model 1, the lowest RMSE of 0.34 mm was for the configuration: learning rate = 0.005, input window = 160, units = 40.

For Model 2, the lowest breathing signal and phase RMSE were 0.12 mm and 8.2°, respectively, for the configuration: learning rate = 0.005, input window = 320, units = 20. The second best configuration resulting in breathing signal and phase RMSE of 0.13 mm and 8.8°, respectively, was: learning rate = 0.01, input window = 160, units = 20.

Model 2 outperformed Model 1. For validation with the patient dataset, the configuration with input window = 160 of Model 2 was used, as it only required 8 s of data to start predicting instead of 16 s (with input window = 320) and had similar performance on the validation dataset.

The mean prediction runtime ± standard deviation of the best two models (both Model 2) on the CPU was 11.2 ms ± 1.2 ms (input window = 160) and 24.9 ms ± 1.9 ms (input window = 320). On the GPU, the mean prediction runtime increased by more than a factor 10 (input window = 160: 164 ms ± 4 ms; input window = 320: 314 ms ± 8 ms) due to memory transfer operations (host memory to GPU and vice versa).

### 3.3 Neural network testing and performance

Table 2 summarizes the results of testing using patient datasets. The average absolute RMSE of the breathing signal for all patients, the ‘large’ and the ‘small’ group was 0.16 mm ± 0.11 mm, 0.33 mm ± 0.07 mm and 0.12 mm ± 0.06 mm, respectively.

**Table 2:** Evaluation of the absolute RMSE for the patient dataset. The RMSE is given for the breathing signal y, the breathing phase φ, the deflection d representing half of a breathing amplitude and the baseline of the breathing signal BL. For reference, the average breathing amplitude of group ‘large’ was 16.2 mm and for group ‘small’ 2.3 mm.

Group	RMSE *y* [mm]	RMSE *φ* [°]	RMSE *d* [mm]	RMSE *BL* [mm]
All (n = 55)	0.16 ± 0.11	14.9 ± 6.8	0.42 ± 0.34	0.26 ± 0.19
‘Large’ amplitude (n = 11)	0.33 ± 0.07	9.5 ± 3.5	0.87 ± 0.29	0.50 ± 0.22
‘Small’ amplitude (n = 44)	0.12 ± 0.06	16.2 ± 6.8	0.31 ± 0.25	0.20 ± 0.12

**Fig. 3:**
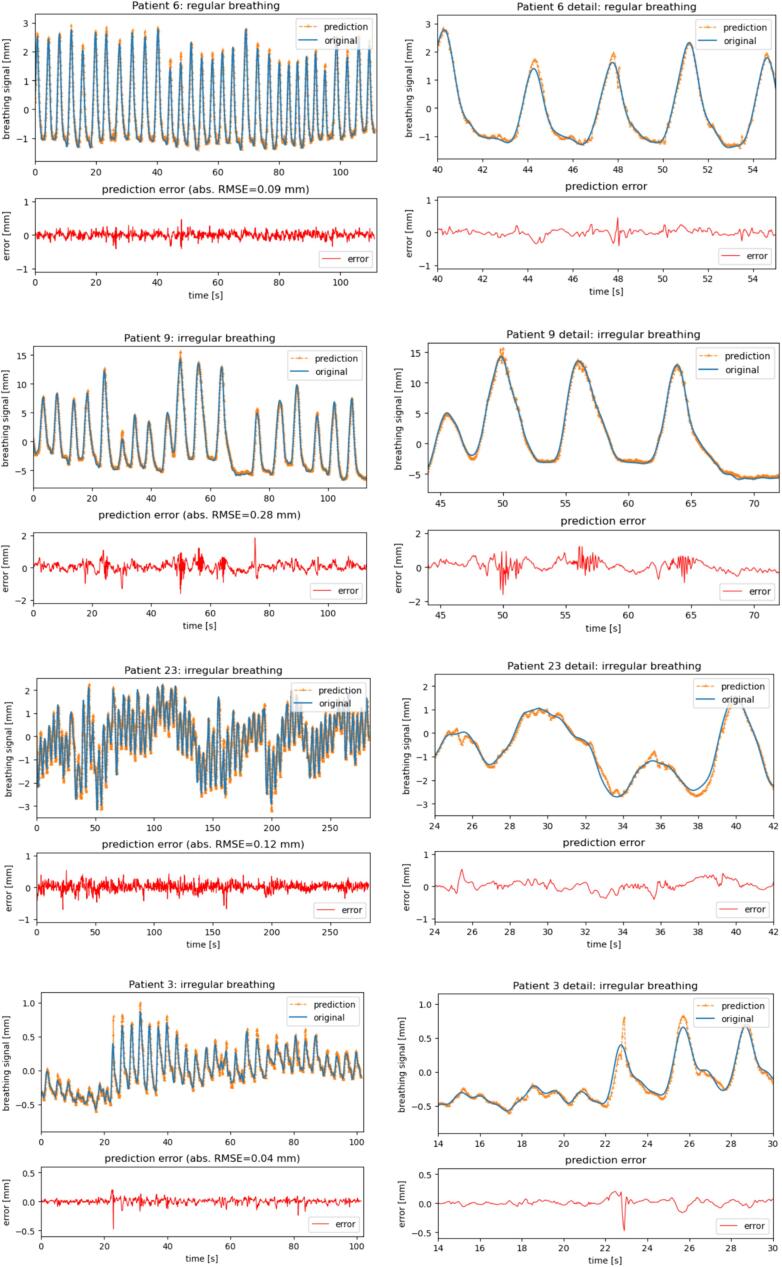
Examples of prediction accuracy for different breathing scenarios. For each example, the whole acquisition is shown in the left column, while the zoom-in is presented in the right column. The original breathing signal is the blue solid line and the predicted breathing signal dashed orange line. Below each breathing signal, the prediction error is given together with the absolute RMSE of the whole breathing data acquisition. Note that the scale of the breathing signal and error can be different due to different amplitudes of an acquisition.

Examples of the prediction accuracy of a ‘regular’ and several ‘irregular’ breathing acquisitions are in Fig. 3. For regular breathing (Patient 6, row 1), the amplitude height prediction was overestimated in some breathing cycles. Patient 9 (row 2) had variations in the amplitude with an expiration break. The expiration break was predicted accurately, however, oscillations in predicted signal were present in some breathing cycles with larger amplitudes. Patient 23 (row 3) had a volatile baseline with amplitude changes, and the model was able to predict these changes. Patient 3 (row 4) had amplitude changes with an unusual breathing pattern. The prediction error for the unusual breathing pattern (14 s to 22 s) was low. However, in the subsequent breathing cycle with much a larger amplitude the amplitude height was overestimated.

## 4 Discussion

The LSTM model trained on a small dataset of breathing signals from healthy volunteers accurately predicted the breathing motion of lung and liver cancer patients with an absolute RMSE of 0.16 mm ± 0.11 mm using a prediction horizon of 500 ms. This supports the translation of motion prediction models into a clinical environment. While collecting a large dataset requires many years [39], the healthy volunteer data can be collected in a very short period of time. Few studies already tried to reduce the amount of data [38], [42], [43], however, never achieved such an accuracy. The implementation of the model is independent of the breathing signal acquisition device. However, model accuracy might be impacted by the temporal resolution of the device.

Data reduction and significant increase in the prediction accuracy was possible due to the decomposition of the breathing signal in model generation (Model 2). The whole procedure including data cleaning and pre-processing together with model training took 17 h on a standard laptop. The validation revealed an absolute RMSE reduction from 0.34 mm (Model 1) to 0.12 mm (Model 2). To our knowledge, this novel approach has not yet been investigated by any other group so far. Additional data size reduction might be possible with data augmentation. This however needs further investigation.

Dynamic scaling and noise filtering helped to characterize the prompt change in respiratory motion and highlighted its intrinsic pattern within a breathing segment [44] eliminating the impact of the absolute amplitude or surface scanner noise. The application of a filter within an input window for the LSTM required additional computational time, however, this time was negligible compared to the prediction horizon of 500 ms. The use of two separate breathing signal locations for the model training enabled an effective doubling of the data and larger diversity. Although the breathing phase was the same for both locations, the amplitude was typically larger with a different breathing pattern in abdomen monitoring [45]. However, it was not possible to conclude which factor, the increased amount of data and/or the increased diversity, mainly contributed to accuracy improvement.

Dividing the dataset into a ‘large’ and a ‘small’ group, revealed differences in the average absolute breathing signal RMSE between the groups (Section 3.3). Given the seven-times larger average breathing amplitude, but only three-times larger average prediction error, the LSTM model had a lower relative prediction error for patients with larger breathing amplitudes. This has two potential reasons: firstly, the share of datasets with ‘large’ amplitude was overrepresented in the training data; secondly, with a larger amplitude the noise of the surface scanner acquisition became less relevant. This provides an important insight on the relevance of amplitude size.

The proposed prediction model offers an essential base for implementing tracking and gating into the radiotherapy workflow as overcoming latencies enables real-time motion mitigation [45]. A breathing motion prediction model could be used for an implementation of *smart* imaging where imaging would be triggered only in a specific phase for tumour position validation instead of continuous tumour monitoring during the whole beam-on time or within defined discrete intervals. This would lead to a reduction of the imaging dose and more efficient imaging. Alternatively, gating at free-breathing would be possible which would increase patient treatment comfort as no breathing guidance would be necessary during the treatment [46]. The implementation of *smart* imaging requires new developments by industry as current systems allow imaging only in fixed time intervals.

The main limitation of our study was the use of surface surrogate signals for tumour motion characterization without providing an integrated internal/external correlation model, i.e. the actual tumour position was not accounted for in the predictions. Several studies revealed already, that the external motion does not directly correspond to the tumour motion [29], [47]. However, our work aimed to demonstrate that motion prediction with a limited amount of data is feasible, independent of the origin of the provided signal. Moreover, only lung and liver cancer patients were used in this study. The applicability for other treatment sites needs further investigation.

In conclusion, our study demonstrated that a motion prediction model can be trained with a low number of healthy volunteers if breathing cycle parameters are implemented. The model achieved highly accurate breathing motion prediction for liver and lung patients. Further research to confirm the generalisability of the model to other tumour sites and the applicability on the internal tumour position prediction is needed.

## CRediT authorship contribution statement

**Andreas Renner:** Conceptualization, Data curation, Investigation, Formal analysis, Methodology, Validation, Visualization, Writing – original draft, Writing – review & editing. **Ingo Gulyas:** Conceptualization, Data curation, Formal analysis, Investigation, Methodology, Software, Writing – original draft. **Martin Buschmann:** Resources, Writing – review & editing. **Gerd Heilemann:** Visualization, Writing – original draft, Writing – review & editing. **Barbara Knäusl:** Resources, Writing – review & editing. **Martin Heilmann:** Resources, Writing – review & editing. **Joachim Widder:** Resources, Writing – review & editing. **Dietmar Georg:** Conceptualization, Funding acquisition, Project administration, Resources, Writing – review & editing. **Petra Trnková:** Conceptualization, Methodology, Supervision, Writing – original draft, Writing – review & editing.

## Declaration of Competing Interest

The authors declare the following financial interests/personal relationships which may be considered as potential competing interests: Barbara Knäusl is associated editor in the journal “Physics and Imaging in Radiation Oncology” and Petra Trnkova member of the editorial board.

## References

[1] Seppenwoolde Y., Shirato H., Kitamura K., Shimizu S., van Herk M., Lebesque J.V. (2002). Precise and real-time measurement of 3D tumor motion in lung due to breathing and heartbeat, measured during radiotherapy. Int J Radiat Oncol Biol Phys.

[2] Yoganathan S.A., Maria Das K.J., Agarwal A., Kumar S. (2017). Magnitude, impact, and management of respiration-induced target motion in radiotherapy treatment: A comprehensive review. J Med Phys.

[3] Meijer K.M., van Dijk I.W.E.M., Frank M., van den Hoek A.D., Balgobind B.V., Janssens G.O. (2023). Diaphragm and abdominal organ motion during radiotherapy: a comprehensive multicenter study in 189 children. Radiat Oncol.

[4] Anastasi G., Bertholet J., Poulsen P., Roggen T., Garibaldi C., Tilly N. (2020). Patterns of practice for adaptive and real-time radiation therapy (POP-ART RT) part I: Intra-fraction breathing motion management. Radiother Oncol.

[5] Zhang Y., Trnkova P., Toshito T., Heijmen B., Aznar M., Albertini F. (2023). A survey of practice patterns for real-time intrafractional motion-management in particle therapy. Phys Imaging Radiat Oncol.

[6] Stroom J.C., Heijmen B.J.M. (2002). Geometrical uncertainties, radiotherapy planning margins, and the ICRU-62 report. Radiother Oncol.

[7] Knopf A.-C., Czerska K., Fracchiolla F., Graeff C., Molinelli S., Rinaldi I. (2022). Clinical necessity of multi-image based (4D(MIB)) optimization for targets affected by respiratory motion and treated with scanned particle therapy - A comprehensive review. Radiother Oncol.

[8] Ehrbar S., Perrin R., Peroni M., Bernatowicz K., Parkel T., Pytko I. (2016). Respiratory motion-management in stereotactic body radiation therapy for lung cancer - A dosimetric comparison in an anthropomorphic lung phantom (LuCa). Radiother Oncol.

[9] Bertholet J., Knopf A., Eiben B., McClelland J., Grimwood A., Harris E. (2019). Real-time intrafraction motion monitoring in external beam radiotherapy. Phys Med Biol.

[10] Buschmann M., Kauer-Dorner D., Konrad S., Georg D., Widder J., Knäusl B. (2024; 200:306-313). Stereoscopic X-ray image and thermo-optical surface guidance for breast cancer radiotherapy in deep inspiration breath-hold. Strahlenther Onkol.

[11] Booth J., Caillet V., Briggs A., Hardcastle N., Angelis G., Jayamanne D. (2021). MLC tracking for lung SABR is feasible, efficient and delivers high-precision target dose and lower normal tissue dose. Radiother Oncol.

[12] Chen G.-P., Tai A., Puckett L., Gore E., Lim S., Keiper T. (2021). Clinical implementation and initial experience of real-time motion tracking with jaws and multileaf collimator during helical tomotherapy delivery. Pract Radiat Oncol.

[13] Uijtewaal P., Borman P.T.S., Woodhead P.L., Hackett S.L., Raaymakers B.W., Fast M.F. (2021). Dosimetric evaluation of MRI-guided multi-leaf collimator tracking and trailing for lung stereotactic body radiation therapy. Med Phys.

[14] Li Y., Li Z., Zhu J., Li B., Shu H., Ge D. (2023). Online prediction for respiratory movement compensation: a patient-specific gating control for MRI-guided radiotherapy. Radiat Oncol.

[15] Tanabe Y., Tanaka H. (2022). Statistical evaluation of the effectiveness of dual amplitude-gated stereotactic body radiotherapy using fiducial markers and lung volume. Phys Imaging Radiat Oncol.

[16] Ehrbar S., Braga Käser S., Chamberlain M., Krayenbühl J., Wilke L., Mayinger M. (2022). MR-guided beam gating: Residual motion, gating efficiency and dose reconstruction for stereotactic treatments of the liver and lung. Radiother Oncol.

[17] Håkansson K., Josipovic M., Ottosson W., Behrens C.P., Vogelius I.R., Persson G. (2023). Evaluating the dosimetric effect of intra-fractional variations in deep inspiration breath-hold radiotherapy - a proof-of-concept study. Acta Oncol.

[18] Aznar M.C., Carrasco de Fez P., Corradini S., Mast M., McNair H., Meattini I. (2023). ESTRO-ACROP guideline: Recommendations on implementation of breath-hold techniques in radiotherapy. Radiother Oncol.

[19] Poulsen P.R., Cho B., Sawant A., Ruan D., Keall P.J. (2010). Detailed analysis of latencies in image-based dynamic MLC tracking. Med Phys.

[20] Rottmann J., Keall P., Berbeco R. (2013). Markerless EPID image guided dynamic multi-leaf collimator tracking for lung tumors. Phys Med Biol.

[21] Bedford J.L., Fast M.F., Nill S., McDonald F.M.A., Ahmed M., Hansen V.N. (2015). Effect of MLC tracking latency on conformal volumetric modulated arc therapy (VMAT) plans in 4D stereotactic lung treatment. Radiother Oncol.

[22] Liu P.Z.Y., Dong B., Nguyen D.T., Ge Y., Hewson E.A., Waddington D.E.J. (2020). First experimental investigation of simultaneously tracking two independently moving targets on an MRI-linac using real-time MRI and MLC tracking. Med Phys.

[23] Worm E.S., Thomsen J.B., Johansen J.G., Poulsen P.R. (2023). A simple method to measure the gating latencies in photon and proton based radiotherapy using a scintillating crystal. Med Phys.

[24] Loebner H.A., Frauchiger D., Mueller S., Guyer G., Mackeprang P.-H., Stampanoni M.F.M. (2023). Technical note: Feasibility of gating for dynamic trajectory radiotherapy - Mechanical accuracy and dosimetric performance. Med Phys.

[25] Jassar H., Tai A., Chen X., Keiper T.D., Paulson E., Lathuilière F. (2023). Real-time motion monitoring using orthogonal cine MRI during MR-guided adaptive radiation therapy for abdominal tumors on 1.5T MR-Linac. Med Phys.

[26] Mann P., Witte M., Mercea P., Nill S., Lang C., Karger C.P. (2020). Feasibility of markerless fluoroscopic real-time tumor detection for adaptive radiotherapy: development and end-to-end testing. Phys Med Biol.

[27] Wikström K.A., Isacsson U.M., Nilsson K.M., Ahnesjö A. (2021). Evaluation of four surface surrogates for modeling lung tumor positions over several fractions in radiotherapy. J Appl Clin Med Phys.

[28] Zhang Y., Huth I., Wegner M., Weber D.C., Lomax A.J. (2017). Surface as a motion surrogate for gated re-scanned pencil beam proton therapy. Phys Med Biol.

[29] Kaestner L., Streb L., Hetjens S., Buergy D., Sihono D.S.K., Fleckenstein J. (2023). Surface guidance compared with ultrasound-based monitoring and diaphragm position in cone-beam computed tomography during abdominal stereotactic radiotherapy in breath-hold. Phys Imaging Radiat Oncol.

[30] Li G. (2022). Advances and potential of optical surface imaging in radiotherapy. Phys Med Biol.

[31] Lu W., Li G., Hong L., Yorke E., Tang X., Mechalakos J.G. (2023). Reproducibility of chestwall and heart position using surface-guided versus RPM-guided DIBH radiotherapy for left breast cancer. J Appl Clin Med Phys.

[32] Freislederer P., Batista V., Öllers M., Buschmann M., Steiner E., Kügele M. (2022). ESTRO-ACROP guideline on surface guided radiation therapy. Radiother Oncol.

[33] Al-Hallaq H.A., Cerviño L., Gutierrez A.N., Havnen-Smith A., Higgins S.A., Kügele M. (2022). AAPM task group report 302: Surface-guided radiotherapy. Med Phys.

[34] Sharp G.C., Jiang S.B., Shimizu S., Shirato H. (2004). Prediction of respiratory tumour motion for real-time image-guided radiotherapy. Phys Med Biol.

[35] Ernst F., Dürichen R., Schlaefer A., Schweikard A. (2013). Evaluating and comparing algorithms for respiratory motion prediction. Phys Med Biol.

[36] Ernst F., Schlaefer A., Schweikard A. (2011). Predicting the outcome of respiratory motion prediction. Med Phys.

[37] Lombardo E., Liu P.Z.Y., Waddington D.E.J., Grover J., Whelan B., Wong E. (2023). Experimental comparison of linear regression and LSTM motion prediction models for MLC-tracking on an MRI-linac. Med Phys.

[38] Wang G., Li Z., Li G., Dai G., Xiao Q., Bai L. (2021). Real-time liver tracking algorithm based on LSTM and SVR networks for use in surface-guided radiation therapy. Radiat Oncol.

[39] Lin H., Shi C., Wang B., Chan M.F., Tang X., Ji W. (2019). Towards real-time respiratory motion prediction based on long short-term memory neural networks. Phys Med Biol.

[40] McNutt T.R., Bowers M., Cheng Z., Han P., Hui X., Moore J. (2018). Practical data collection and extraction for big data applications in radiotherapy. Med Phys.

[41] Li G., Wu X., Ma X. (2022). Artificial intelligence in radiotherapy. Semin Cancer Biol.

[42] Lombardo E., Rabe M., Xiong Y., Nierer L., Cusumano D., Placidi L. (2022). Offline and online LSTM networks for respiratory motion prediction in MR-guided radiotherapy. Phys Med Biol.

[43] Lombardo E., Rabe M., Xiong Y., Nierer L., Cusumano D., Placidi L. (2023). Evaluation of real-time tumor contour prediction using LSTM networks for MR-guided radiotherapy. Radiother Oncol.

[44] Ruan D., Fessler J.A., Balter J.M., Keall P.J. (2009). Real-time profiling of respiratory motion: baseline drift, frequency variation and fundamental pattern change. Phys Med Biol.

[45] Dhont J., Harden S.V., Chee L.Y.S., Aitken K., Hanna G.G., Bertholet J. (2020). Image-guided Radiotherapy to Manage Respiratory Motion: Lung and Liver. Clin Oncol.

[46] Hickling S.V., Veres A.J., Moseley D.J., Grams M.P. (2021). Implementation of free breathing respiratory amplitude-gated treatments. J Appl Clin Med Phys.

[47] Wang G., Song X., Li G., Duan L., Li Z., Dai G. (2022). Correlation of optical surface respiratory motion signal and internal lung and liver tumor motion: a retrospective single-center observational study. Technol Cancer Res Treat.

