# Corrigendum to “Explicitly encoding the cyclic nature of breathing signal allows for accurate breathing motion prediction in radiotherapy with minimal training data” [Phys. Imaging Radiat. Oncol. 30 (2024) 100594]

**DOI:** 10.1016/j.phro.2025.100718

**Published:** 2025-02-01

**Authors:** Andreas Renner, Ingo Gulyas, Martin Buschmann, Gerd Heilemann, Barbara Knäusl, Martin Heilmann, Joachim Widder, Dietmar Georg, Petra Trnková

**Affiliations:** a Department of Radiation Oncology Medical University of Vienna Vienna Austria; b Christian Doppler Laboratory for Image and Knowledge Driven Precision Radiation Oncology Department of Radiation Oncology Medical University of Vienna Austria; c MedAustron Ion Therapy Center Wiener Neustadt Austria

The authors of this article would like to provide further clarification of a sentence that appeared in section 2.2 of the published version.

In *Material and Methods,* in Section 2.2*. Data pre-processing,* we described the model which explicitly encodes the cyclic nature of the breathing signal (denoted as *Model 2* – and the conventional model for comparison as *Model 1*). The description of Model 2 was directly followed by describing the process of *dynamic scaling* without explicitly mentioning that the *dynamic scaling* was only used for *Model 2*. This needs to be clarified by adding “Model 2” to the first sentence of the 4th paragraph of Section 2.2.

The correct version of the sentence should read: “Dynamic scaling was used for pre-processing of training data **of Model 2** to enhance the influence of each breathing pattern segment, which was transformed by the scaling step into the range [−1,1].”

The authors apologise for this oversight.

